# Maternal satisfaction about childhood immunization in primary health care center, Egypt

**DOI:** 10.11604/pamj.2014.18.157.1773

**Published:** 2014-06-18

**Authors:** Hanan Abbas Abdo Abdel Rahman El Gammal

**Affiliations:** 1 Family Medicine Department, Faculty Of Medicine, Suez Canal University

**Keywords:** Childhood immunization, maternal knowledge, maternal satisfaction

## Abstract

**Introduction:**

Childhood immunization is considered to be among the most effective preventive services, and is therefore critical to monitor and evaluate. One prior study reported an association between parental satisfactions with pediatric care and up-to-date immunization at 24 months independent of maternal age, race, and education. In addition to promoting appropriate utilization, satisfaction may increase engagement in the health care process. Health system factors included inconvenient clinic hours, dates or locations, waiting lines, and conflicting information. The inconvenience of clinic hours dates of immunization clinics, and locations of clinics were reported by 75% of the parents.

**Methods:**

A cross section study was conducted on three hundred and thirty five mothers chosen from PHCC participating in the study by providing information on satisfaction about the program and their knowledge about vaccination

**Results:**

Inappropriate knowledge was reported by most of mothers (84.8%). And 95.2% of mothers were satisfied with childhood immunization services in primary healthcare center, compared to 4.8% who were unsatisfied with them.

**Conclusion:**

This study shows that there was no statistically significant relation between maternal satisfaction with childhood immunization services and knowledge score, while in most satisfaction surveys information giving was an important need and this represent that client needs are changing, and priorities from client's perspectives are also changing, so on- going monitoring of client satisfaction is the safeguard to improve quality of care.

## Introduction

The World Health Organization (WHO) identified the need for public health intervention and in 1974 initiated the Expanded Programme of Immunization (EPI), which aims to immunize, and thus protect, mothers against Tetanus and their children against the following six deadly diseases: Childhood Tuberculosis, Poliomyelitis, Diphtheria, Pertussis, Measles and Tetanus [1].

Childhood immunization is considered to be among the most effective preventive services, and is therefore critical to monitor and evaluate [2]. Timely immunization depends on both appropriate access to health care and the administration of eligible doses at each visit [3].

Immunization remains one of the most important public health interventions and a cost effective strategy to reduce both the morbidity and mortality associated with infectious diseases. Over two million deaths are delayed through immunization each year worldwide [4]. Despite this, vaccine preventable diseases remain the most common cause of childhood mortality with an estimated three million deaths each year [5]. Uptake of vaccination services is dependent not only on provision of these services but also on other factors including knowledge and attitude of mothers, density of health workers [6].

Parental satisfaction with pediatric care is an indicator of provider quality that has been relatively unexplored in relation to childhood immunization. One prior study reported an association between parental satisfactions with pediatric care and up-to-date immunization at 24 months independent of maternal age, race, and education [7]. Research on parental health beliefs and attitudes often assumes that parents decline immunization or are simply less knowledgeable and persistent in the health care setting without also examining their access and timely utilization of well-child care [8].

Assessing outcomes has merit both as an indicator of the effectiveness of different interventions and as part of a monitoring system directed to improving quality of care as well as detecting its deterioration [9].

For client satisfaction, clients are asked to assess not their own health status after receiving care but their satisfaction with the services delivered [10, 11]. Some studies found that Consumers′ satisfaction with health care services in Africa was one of the most important factors determining the utilization of services [12–16]. Determinants of perceptions of quality of services found in Tanzania Include; perceived time spent at the facility, availability of immunizations, availability of child health services and the staff strength of the health facilities [17].

The inconvenience of clinic hours dates of immunization clinics, and locations of clinics were reported by 75% of the parents [4].

A satisfied patient is more likely to develop a deeper and long-lasting relationship with their medical provider, leading to improved compliance, continuity of care, and ultimately better health care outcomes [18].

Research on parental health beliefs and attitudes often assumes that parents decline immunization or are simply less knowledgeable and persistent in the health care setting without examining their access and utilization of well-child care [19–21].

In past researches, lack of motivation on the part of EPI staff, absence of vaccinators, inconvenient locations and problems with the cold chain have been cited as common reasons for obstruction of immunization [22–25].

Education of mothers was identified as a major factor for increased immunization of Nigerian children in a rural area [25].

Immunization uptake in the Republic of Ireland remains below the World Health Organization target. Studies examining the maternal aspects of this phenomenon have established the following factors as contributing to suboptimal uptake: rising parity, low knowledge regarding immunization, particularly the timing of the next due vaccine, inadequate antenatal care (probably representing poor engagement with medical services), family dysfunction and inadequate social support. Studies have found that the fear of vaccine harm was a barrier to immunization.

Mothers point to long waiting times and inconvenient hours. Qualitative studies point additionally to difficulty obtaining an appointment, and crowded clinics [26].

## Methods

In this study the knowledge score of the patient was assessed, then maternal satisfaction was assessed and study its relationship to knowledge score. The study sample included all 335 mothers who were attending primary care center for vaccination of children less than 2 years of age between September 2010 and May 2011, completed an interview about knowledge about vaccination and satisfaction about the service.

### Data sources

Three data sources inform these analyses: a maternal questionnaire, consisted from a brief questionnaire provided socio-demographic data about mother, knowledge about vaccination from the mother, and regarding services received and satisfaction with services (Appendix). The interviews were conducted in Arabic by trained interviewers about immunizations and maternal satisfaction about vaccination services.

### Study Variables

Maternal knowledge was assessed by a knowledge score questionnaire, and maternal satisfaction with vaccination was evaluated using a global measure assessment tool. Mothers were asked to rate doctors and nurses at their site of care in providing good health care with responses grouped as excellent, good, or fair/poor. This measure modified from the global measure included in Consumer Assessment of Health Plan Satisfaction, which asks mothers to rate their child's health care on a scale from 0 to 5 (10 indicating the best possible care) including questions about information giving, waiting time, waiting place, staff attitude, affordability of vaccination.

### Setting

This study was conducted at primary care unit affiliated to ministry of health and population (MOHP), at the vaccination area at urban primary health care center.

**Analysis** The data was collected by the researcher and analyzed by using SPSS V. 14, to assess knowledge score, and assess satisfaction level, and relation between satisfaction and knowledge was assessed by using chi square test.

**Ethical considerations:** The study was approved by the ethics committee of the faculty of medicine, suez canal university, and has been performed in accordance with the ethical standards laid down In the declaration of Helsinki(1964). Questionnaire was anonymous and didn't contain any critical questions, and confidentiality of the data were maintained.

## Results

This study shows that the majority of mothers (91.9%) aged 20 years or more. Intermediate education (Primary/Secondary) represented 54.6% of mothers compared to 33.7% for high education (University education), 8.1% can read & write and only 8.1% are illiterate mothers. On the other hand, 63.3% of mothers had only one-to-two children compared to 28.1% who had three-to-four children and only 8.6% had more than four children (Table 1).

**Table 1:** Distribution of mothers according to Socio-demographic characteristics

Socio-demographic characteristics	Frequency (n = 335)	
	No.	%
**Maternal Age**		
< 20 years	27	8.1
≥ 20 years	308	91.9
**Educational Level**		
Illiterate	12	3.6
Read & Write (barely)	27	8.1
Primary/Secondary	183	54.6
University educational	113	33.7
**Number of Children**		
1-2	212	63.3
3-4	94	28.1
5-6	29	8.6

The present study shows that inappropriate knowledge was reported by most of mothers (84.8%), compared to 14.9% for fair knowledge and only 0.3% for good knowledge (Table 2).

**Table 2:** Distribution of mothers according to their knowledge scores

Knowledge Score	Frequency	
	No.	%
Inappropriate	284	84.8
Fair	50	14.9
Good	1	0.3
Total	**335**	**100.0**

This study shows that there was no statistically significant relation between vaccination coverage and maternal knowledge. The majority of those with full vaccination coverage (84.5%) and all of those with deficient vaccination coverage had inappropriate knowledge (Table 3).

**Table 3:** Relation between knowledge scores and vaccination coverage

Knowledge Score	Vaccination Coverage		P-Value
	Full No. (%)	Deficient No. (%)	
Inappropriate	279 (84.5)	5 (100.0)	> 0.05
Appropriate ^a^	51 (15.6)	0	
**Total**	**330 (100.0)**	**5 (100.0)**	

In the present study 57.3% of mothers evaluated childhood immunization services as good compared to 40.6% of mothers who evaluated it as fair, while 2.1% evaluated it as inappropriate (Table 4).

**Table 4:** Distribution of mothers according to their evaluation of immunization services

Service evaluation	Frequency (n = 335)	
	No.	%
Inappropriate	7	2.1
Fair	136	40.6
Good	192	57.3
Total	**335**	**100.0**

In this study The results show that 57.3% of mothers evaluated childhood immunization services as good compared to 40.6% of mothers who evaluated it as fair, while 2.1% evaluated it as inappropriate.

The results show that 95.2% 0f mothers were satisfied with childhood immunization services in primary healthcare center, compared to 4.8% who were unsatisfied with them.

Maternal satisfaction about staff attitude was 66.7%, satisfaction about waiting place was 62.9%, satisfaction about waiting time 61.5%, satisfaction about information giving was 61%, and satisfaction about cost was 50.5%.

This study represents that there was no statistically significant relation between maternal satisfaction with childhood immunization services and knowledge score; p > 0.05. However, this relation shows the majority of satisfied mothers had inappropriate knowledge (85.3%) (Table 5).

**Table 5:** Relation between maternal satisfaction with immunization services and their knowledge score

Knowledge Score	Maternal Satisfaction with immunization services		*P*-Value
	Unsatisfied No. (%)	Satisfied No. (%)	
Inappropriate	12 (75.0%)	272 (85.3%)	0.265
Fair/ Good	4 (25.0%)	47 (14.7%)	
**Total**	**16** (100.0)	**319** (100.0)	

## Discussion

This study is a cross-sectional study which involved 335 mothers attending primary health care center in Ismailia Governorate for immunization of their children, aiming to study maternal knowledge, and its relation to satisfaction about vaccination services. In this study the majority of those mothers 91.9% aged 20 years or more. Intermediate education (Primary/Secondary) represented 54.6% of mothers compared to 33.7% for high education (University education), 8.1% for read & write and only 8.1% were illiterate mothers.

A study of Immunization-related knowledge, attitudes and practices of mothers; done on Congo found that mothers aged more than 20 years were 95% which is in agreement with this study which the educational level showed that Intermediate education (Primary/Secondary) represented 81.6% of mothers compared to 16.2% for high education (University education), 2.2% for illiterate mothers [27]. This could be due to better care about education in Egypt is more than Congo.

In the present study inappropriate knowledge was reported by most of mothers 84.8%, compared to 14.9% for fair knowledge and only 0.3% for good knowledge. While in The study of Knowledge and perception of mother with children less than 2 years of age conducted in Lao PDR revealed that 60.5% of mother had adequate knowledge and 39.5% had inadequate knowledge [28]. This difference could be due to that although mothers having inappropriate knowledge and don't care about type of vaccine or diseases will it prevent, but they only care about her child took the vaccine as a perceived importance of complications and disability related to non vaccination.

There was no statistically significant relation between vaccination coverage and maternal knowledge. The majority of those with full vaccination coverage 84.5% and 100% of those with deficient vaccination coverage had inappropriate knowledge, which is different from the study of Knowledge and attitude on immunization preventable diseases of mother with children 6-24 month old and completeness of their children's immunization done in Thailand. It showed that 82.4% of mothers who had adequate knowledge have their children completely immunized while only 56% of mothers with inadequate knowledge had their children completely immunized. There was no association between the knowledge and completeness of immunization [29–32]. This could be explained that the whole maternal satisfaction about quality have more than one element other than knowledge such as doctor pt relationship, staff attitude, cost, and waiting time, and despite low knowledge score but remains the fact that source of information when it is given by a physician is better than other source from client perspective, and the only motive to attend for vaccination program is the maternal perceived seriousness of the disease.

The present study showed that the vaccination services rendered by the centre had succeeded in generating satisfaction among majority of the studied sample. The low level of satisfaction in the others may be due to influence of certain Socio-cultural and demographic factors. With increasing education one′s expectation increases, which may explain the less satisfaction among the more highly educated [33]. Apart from the differences due to these variables, the centre seems to have provided good quality of services so as to have achieved excellent or good level of satisfaction among majority of mothers served. The deficiency that is still remaining may be overcome by generating high level of knowledge and awareness among the mothers by increase awareness mothers′ meetings and extensive campaigns, inviting opinions and suggestions from the mothers and encouraging maternal active participation.

## Conclusion

Maternal satisfaction about vaccination is crucial to completeness of the schedule but it doesn′t depend mainly on maternal knowledge about vaccination but other factors such as staff attitude, waiting time and cost of the service.

Despite vaccination coverage is very high and maternal satisfaction is at a good level, but still there is a window for improvement in source of information to involve the physician more often in the information giving process.

## References

[1] Bates AS, Wolinsky FD (1998). Personal, financial, and structural barriers to immunization in socio economically disadvantaged urban children. Pediatrics..

[2] Prislin R, Dyer JA, Blakely CH, Johnson CD (1998). Immunization status and sociodemographic characteristics: the mediating role of beliefs, attitudes, and perceived control. Am J Public Health..

[3] Rosenstock IM (1999). Historical origin of the Health Belief Model. Health Educ Monogr..

[4] Luman ET, McCauley MM, Shefer A, Chu SY (2003). Maternal characteristics associated with vaccination of young children. Pediatrics..

[5] World Health Organization (WHO) Immunization, vaccines and biologicals: Centre for Global Development: Making Markets for vaccines: from ideas to actions.

[6] Matsumura T, Nakayama T, Okamoto S, Ito H (2005). Measles vaccine coverage and factors related to uncompleted vaccination among 18-month-old and 36-month-old children in Kyoto, Japan. BMC Public Health..

[7] Torun SD, Bakirci N (2006). Vaccination coverage and reasons for non-vaccination in a district of Istanbul. BMC Public Health..

[8] Anand S, Bärnighausen T (2007). Health workers and vaccination coverage in developing countries: an econometric analysis. Lancet..

[9] Gust DA, Strine TW, Maurice E (2004). Underimmunization among children: effectsof vaccine safety concerns on immunization status. Pediatrics..

[10] Bennett P, Smith C (1992). Parents attitudinal and social influences on childhood vaccination. Health Educ Res..

[11] Epstein A (1990). Sounding board: the outcomes movement, will it get us where we want to go. New Engl J Med..

[12] Blumenfeld SN (1993). Quality assurance in transition. PapuaNew Guinea Med J..

[13] Fisher AW (1971). Patient's evaluation of outpatient medical care. J Med Educ..

[14] Barnett B (1995). Women's views influence choice. Network..

[15] Akin JS, Hutchinson P (1999). Health-care facility choice and the phenomenon of by-passing. Hlth Policy Plann..

[16] Masatu MC, Klepp KI, Kvale G (2001). Use of health services and reported satisfaction among primary school adolescents in Arusha, Tanzania. J Adol Hlth..

[17] Malata M (2000). First-time mothers’ satisfaction with labour and childbirth information received: a Malawian perspective. Clin Excellence in Nursing Practice..

[18] Whitworth J, Pickering H, Mulwanyi F (1999). Deterrninants of attendance and patient satisfaction at eye clinics in south-western Uganda. Hlth Policy and Plann..

[19] Newman RD, Gloyd S, Nyangezi JM, Machobo F, Muiser J (1998). Satisfaction with outpatient health care services in Manica Province, Mozambique. Hlth Policy Plann..

[20] Speizer IS, Bollen KA (2000). How well do perceptions of family planning service quality correspond to objective measures: Evidence from Tanzania. Stud Family Plann..

[21] Prislin R, Dyer JA, Blakely CH, Johnson CD (1998). Immunization status and sociodemographic characteristics: the mediating role of beliefs, attitudes, and perceived control. Am J Public Health.

[22] Gust DA, Strine TW, Maurice E (2004). Under immunization among children: effects of vaccine safety concerns on immunization status. Pediatrics..

[23] Bennett P, Smith C (1992). Parents attitudinal and social influences on childhood vaccination. Health Educ Res..

[24] Ahmad N, Akhtar T, Roghani MT, Ilyas HM, Ahmad M (1999). Immunization coverage in three districts of North West Frontier Province (NWFP). J Pak Med Assoc..

[25] Provincial EPI Directorate (2000). Expanded Programme of Immunization: Logistics Inventory.

[26] Odusanya OO, Alufohai EF, Meurice FP, Ahonkhai VI (2008). Determinants of vaccination coverage in rural Nigeria. BMC Public Health..

[27] Peter M Harrington, Catherine Woodman, William F Shannon (2000). Low immunisation uptake: Is the process the problem. J Epidemiol Community Health.

[28] Williams SJ, Calnan M (1991). Key determinants of consumer satisfaction with general practice. Fam Pract..

[29] Mapatano MA (2008). Immunization-related knowledge, attitudes and practices of mothers in Kinshasa, Democratic Republic of the Congo. Fam Pract SA..

[30] Saleumsak K (2002). Knowledge and perception of mother with children under 2 years of age on immunization status in Khammeuan province.

[31] Phounphenghack K (2007). Knowledge and perception of mother about immunization of children under 3 years of age in the Saythany Distrect, Vientiane LAO PDR.

[32] Vongkhamdy K (2005). Knowledge and attitude on immunization preventable diseases of mother with children 6-24 month old and completeness of their children's immunization in Pakse district, Champasake province, Lao PDR.

[33] Banerjee B (2003). A qualitative analysis of maternal and child health services of an urban health center, by assessing client perception in terms of awareness, satisfaction and service utilization. Indian Journal of Community Medicine..

